# *In-Vivo* Quantitative Image Analysis of Age-Related Morphological Changes of *C. elegans* Neurons Reveals a Correlation between Neurite Bending and Novel Neurite Outgrowths

**DOI:** 10.1523/ENEURO.0014-19.2019

**Published:** 2019-07-08

**Authors:** Max Hess, Alvaro Gomariz, Orcun Goksel, Collin Y. Ewald

**Affiliations:** 1 Eidgenössische Technische Hochschule Zürich, Department of Health Sciences and Technology, Institute of Translational Medicine, Schwerzenbach-Zürich CH-8603, Switzerland; 2 Eidgenössische Technische Hochschule Zürich, Department of Information Technology and Electrical Engineering, Computer-Assisted Applications in Medicine Group, Zürich, CH-8092, Switzerland

**Keywords:** aging, *C. elegans*, heterogeneity, morphology, neurite bending, neurite outgrowth

## Abstract

The aging of the human brain in the absence of diseases is accompanied by subtle changes of neuronal morphology, such as dendrite restructuring, neuronal sprouting, and synaptic deteriorations, rather than neurodegeneration or gross deterioration. Similarly, the nervous system of *Caenorhabditis elegans* does not show neurodegeneration or gross deterioration during normal aging, but displays subtle alterations in neuronal morphology. The occurrence of these age-dependent abnormalities is stochastic and dynamic, which poses a major challenge to fully capture them for quantitative comparison. Here, we developed a semi-automated pipeline for quantitative image analysis of these features during aging. We employed and evaluated this pipeline herein to reproduce findings from previous studies using visual inspection of neuronal morphology. Importantly, our approach can also quantify additional features, such as soma volume, the length of neurite outgrowths, and their location along the aged neuron. We found that, during aging, the soma of neurons decreases in volume, whereas the number and length of neurite outgrowths from the soma both increase. Long-lived animals showed less decrease in soma volume, fewer and shorter neurite outgrowths, and protection against abnormal sharp bends preferentially localized at the distal part of the dendrites during aging. We found a correlation of sharp bends with neurite outgrowth, suggesting the hypothesis that sharp bends might proceed neurite outgrowths. Thus, our semi-automated pipeline can help researchers to obtain and analyze quantitative datasets of this stochastic process for comparison across genotypes and to identify correlations to facilitate the generation of novel hypothesis.

## Significance Statement

The etiology of age-dependent morphologic changes in neurons remains elusive. The heterogeneity of these refinements requires an unbiased quantitative acquisition and analysis to pin-point the molecular underpinning of these structural changes. Here, we developed a work-flow and adopted algorithms to allow researchers to capture and analyze these age-dependent changes of *Caenorhabditis elegans* touch receptor neurons *in vivo*. Increasing the traceability and quantification of these stochastic changes will aid researchers to gain mechanistic insights into the underlying biology of these age-dependent morphologic changes in aging neurons.

## Introduction

Aging is accompanied by a gradual impairment of sensory and motor function and cognitive decline, thereby lowering the quality of life of elderly people (Yankner et al., 2008; Partridge et al., 2018). Neurodegenerative diseases, such as Alzheimer’s disease, are among the top ten causes of death (Alzheimer's Association, 2018) and are characterized by improper protein aggregation and neuronal vulnerability for synapse loss, neuronal apoptosis, and white matter degeneration (Yankner et al., 2008). By contrast, during healthy aging most neurons are remarkably preserved in the human brain (Freeman et al., 2008). Aging of the cortical neuronal regions of the human brain are associated with altered cell-cell interactions and subtle morphologic changes, such as neurite sprouting, rather than neuronal loss (Burke and Barnes, 2006; Yankner et al., 2008; Fjell and Walhovd, 2010). Thus, dendrite restructuring, neuronal sprouting, and synaptic deterioration are thought to underlie the cognitive functional decline of the human brain during aging. The underlying biology of these subtle morphologic changes of aging neurons remain poorly understood.

The nematode *Caenorhabditis elegans* with its short lifespan of three weeks and stereotypic and anatomically invariant architecture of 302 neurons provides a powerful *in-vivo* model to study these age-related morphologic features (Chen et al., 2013; Chew et al., 2013a; Bénard and Doitsidou, 2016). Age-associated changes have been observed in *C. elegans* touch receptor neurons (Pan et al., 2011; Tank et al., 2011; Toth et al., 2012), dopaminergic neurons (Toth et al., 2012), GABAergic neurons (Tank et al., 2011), and cholinergic ventral cord neurons (Pan et al., 2011) under electron microscopy imaging of wild-type (WT) *C. elegans* or by using transgenic GFP expression to outline neuronal shape. These morphologic abnormalities start in young adult *C. elegans* (day 4 of adulthood) when animals are still in their reproductive phase and become progressively worse during aging (Bénard and Doitsidou, 2016).

**Figure 1.**
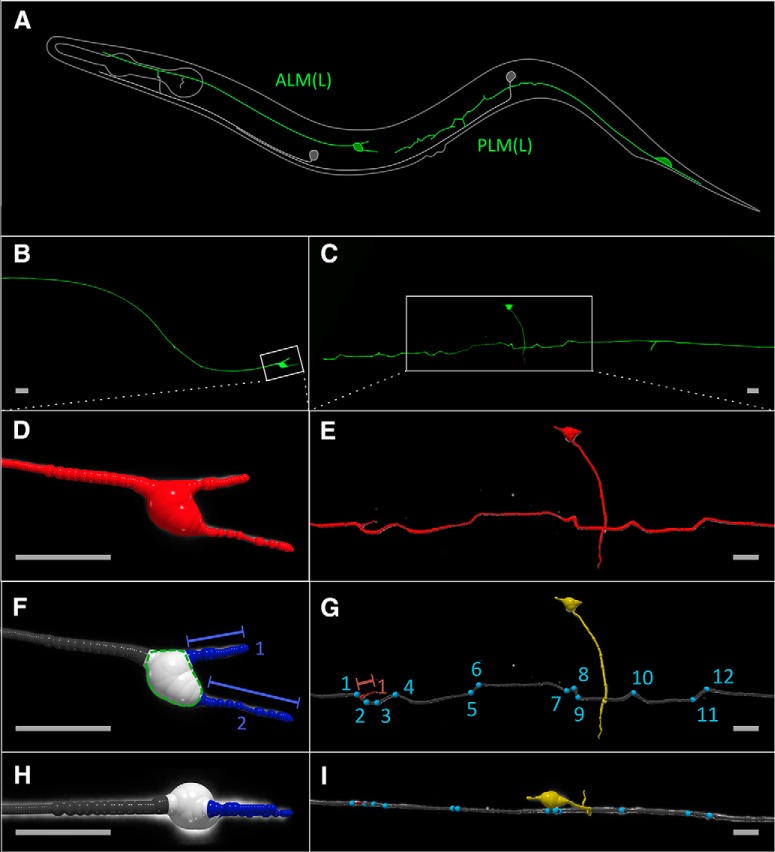
Visual representation of the neuronal tracing algorithm and the confocal image of the *C. elegans* touch receptor neurons. ***A***, Overview of *C. elegans* ALM and PLM touch receptor neurons. Only the left ALM(L) and PLM(L) neurons are shown in green. For detailed workflow for image acquisition and processing of ALM and PLM neuronal morphology see Extended Data Figure 1-1. ***B***, Z-projection of a typical ALM neuron at day 8 of adulthood. ***C***, Z-projection of a typical PLM neuron at day 8 of adulthood. ***D***, ***E***, Output of the APP2 neuron-tracing algorithm of region of interest shown in ***B***, ***C***, respectively (white rectangles), consists of a tree structure of connected nodes shown in red. ***F***, ***H***, Classified ALM neuronal tree with main-branch (gray), soma-nodes (white) and soma outgrowth (blue) in frontal (***F***) and top (***H***) view. Quantified morphologic features (soma-volume, soma outgrowth count and soma outgrowth length) are indicated in (***F***) and corresponding ground truth is shown in Extended Data Figure 1-2. ***G–I***, Classified PLM tree with main-branch (gray), neurite-outgrowth (red) crossing PVM neuron (yellow) and sharp bends (blue) in lateral (***G***) and dorsal (***I***) view. Quantified morphologic features (bend count, neurite-outgrowth count and neurite-outgrowth length) are indicated in ***G*** and corresponding ground truth and quantification of sharp bends are shown in Extended Data Figures 1-2, 1-3. The heterogeneity of these age-related morphologic changes is illustrated by a collage of randomly selected z-projection of these neurons depicted in Extended Data Figure 1-4. Scale bars = 10 μm.*Figure Contributions:* Max Hess made all the figures.

The neurons that show the most striking age-related morphologic abnormalities are the touch receptor neurons (ALML, ALMR, AVM, PLML, PLMR, PVM; Chalfie and Sulston, 1981; Goodman, 2006; Chen et al., 2013; Chew et al., 2013a). Touch receptor neurons are surrounded by a special extracellular matrix and are attached to the hypodermis and cuticle (Chalfie and Sulston, 1981). In response to gentle touch, the touch receptor neurons sense and signal via interneurons to the motor neurons to initiate either a forward or backward movement of *C. elegans* (Chalfie and Sulston, 1981; Goodman, 2006). The touch receptor neurons are non-ciliated mechanosensory neurons, which extend long dendrites that span across either the anterior or posterior half of *C. elegans* (Fig. 1*A*; Chalfie and Sulston, 1981). These dendrites are filled with unusual 15 protofilament microtubules (Goodman, 2006). Neuronal microtubule-associated protein PTL-1, which is the orthologue of human Tau, together with β-spectrin (UNC-70) are required for maintaining touch receptor neuron shape to withstand mechanical stress (Krieg et al., 2017). Loss of shortens lifespan and accelerates the progression of morphologic abnormalities of these touch receptor neurons (Chew et al., 2013b). Expressing human Tau in the six touch receptor neurons results in morphologic abnormalities (Miyasaka et al., 2005). Furthermore, overexpression of the *C. elegans* APL-1, the orthologue of human amyloid precursor protein (APP), in the six touch receptor neurons results in an impairment of touch habituation (Ewald et al., 2012) and in shorter lifespan (Ewald et al., 2016). Thus, loss of the integrity of these six touch receptor neurons is not only important for behavioral plasticity, but also has far-reaching consequences affecting organismal aging and lifespan.

10.1523/ENEURO.0014-19.2019.f1-1 Extended Data Figure 1-1 Workflow of the processing pipeline. ***A***, Schematic representation of four out of six touch receptor neurons of *C. elegans*. ALM and PLM neurons that were used for image processing are shown in green. ***B***, Three confocal image stacks were recorded per *C. elegans* (blue squares; ***A***) leading to raw image stacks. ***C***, Image stacks were pre-processed by stitching, gaussian blur, and manual removal of artefacts that could interfere with neuron tracing. ***D****–****F***, A neuron tracing algorithm (APP2) was used to generate three-dimensional neuronal trees consisting of connected nodes (***D***), which were classified (***E***), and the morphology was quantified (***E***, ***F***) using a semi-automated pipeline generated in this study. For a more detailed description, please see Materials and Methods. Download Figure 1-1, EPS file.

**Figure 2.**
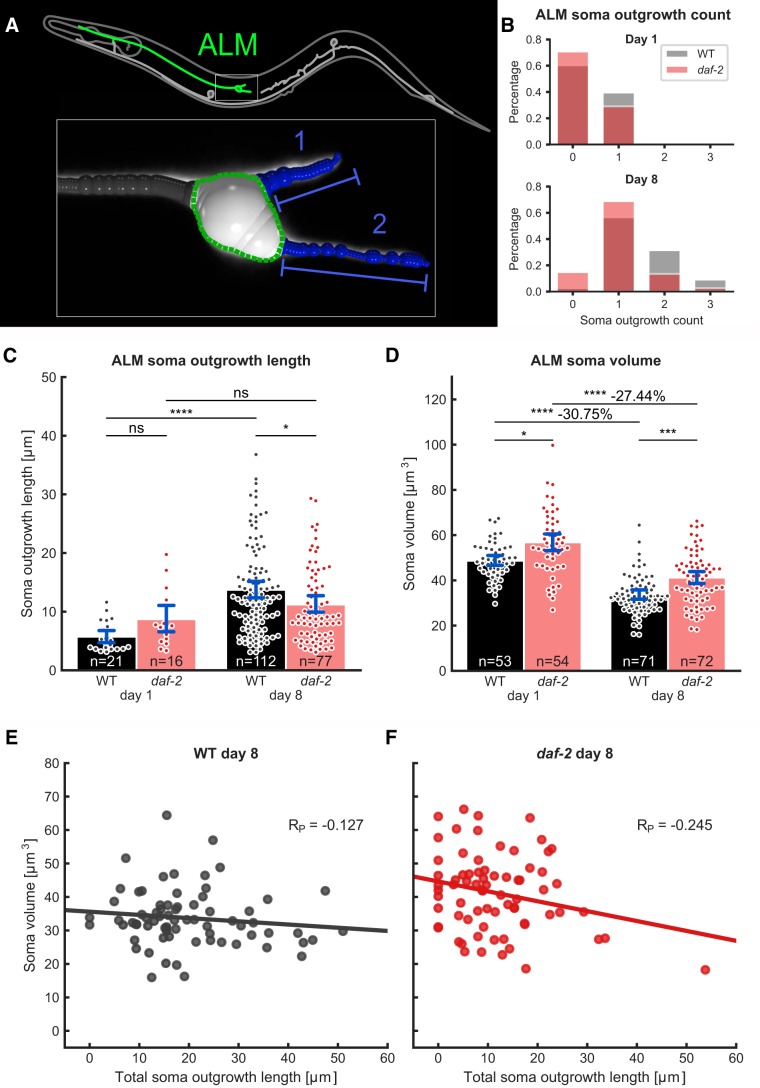
The ALM neuron soma volume and the number and length of soma outgrowths are less effected by aging in long-lived *C. elegans*. ***A***, Overview of quantified morphologic features in ALM neurons. Inset shows enlarged soma region with quantifications indicated (soma outgrowth count and length, blue; soma-volume, green). ***B***, Soma outgrowth counts on days 1 and 8 of adulthood for WT and *daf-2(e1370)* mutants. Both conditions show a significant increase in soma outgrowth counts during aging (WT, *p* = 3.17e-15; *daf-2(e1370)*, *p* = 1.94e-09). There is no significant difference between genotypes at day 1 (*p* = 0.376), but at day 8, soma outgrowth counts are significantly lower for *daf-2(e1370)* mutants compared to WT (*p* = 2.08e-03). For direct comparison of this data with previous studies, we replotted this data in percentage of ALM neurons with soma outgrowths (Extended Data Fig. 2-1). ***C***, Lengths of individual soma outgrowths increase with age. This effect is statistically significant in WT animals (*p* = 2.20e-07), but no statistical significance is reached for *daf-2(e1370)* mutants (*p* = 0.149). At day 8 of adulthood, WT animals have significantly longer soma outgrowths (*p* = 3.38e-02). Error bars indicate bootstrapped 95% confidence interval of the mean, *n* is the number of outgrowth events scored. ***D***, ALM soma volume decreases between day 1 and day 8 of adulthood (WT, *p* = 9.55e-12; *daf-2(e1370)*, *p* = 1.52e-08), whereas WT somas are smaller than *daf-2(e1370)* somas (day 1, *p* = 3.60e-02; day 8, *p* = 2.44e-04). Error bars indicate bootstrapped 95% confidence interval of the mean, n is the number of neurons scored. ***E***, ***F***, Correlation of total soma outgrowth length (lengths of all soma outgrowths summed) versus soma volume of day 8 of adulthood *C. elegans* does not show a strong correlation for both WT (***E***, Pearson’s *R* = –0.127) and *daf-2(e1370)* mutants (***F***, Pearson’s *R* = –0.245). Data were collected in three independent trials and pooled for the analysis shown in this figure (***B***–***F***). ns = *p* > 0.05, * = *p* < 0.05, *** = *p* < 0.001, **** = *p* < 0.0001.*Figure Contributions:* Max Hess made all the figures.

10.1523/ENEURO.0014-19.2019.f1-2 Extended Data Figure 1-2 Ground truth validation comparing automated results with manual and visually observed results. ***A***, ***B***, Pair-wise plot (***A***) and scatter-plot (***B***) of soma outgrowth lengths in automatic measurements and manually traced branches using the ImageJ “Simple neurite tracer” plug-in. ***C***, ***D***, Pair-wise plot (***C***) and scatter-plot (***D***) of soma volumes measured automatically and manually segmented soma. R_p_ is Pearson’s correlation coefficient. ***E***, Bend count for 12 PLM neurons evaluated by seven people manually (black, thin), mean of manual counts (black, thick) and automatic counts (green). Algorithmic counts are close to the mean of manual counts. Download Figure 1-2, EPS file.

**Figure 3.**
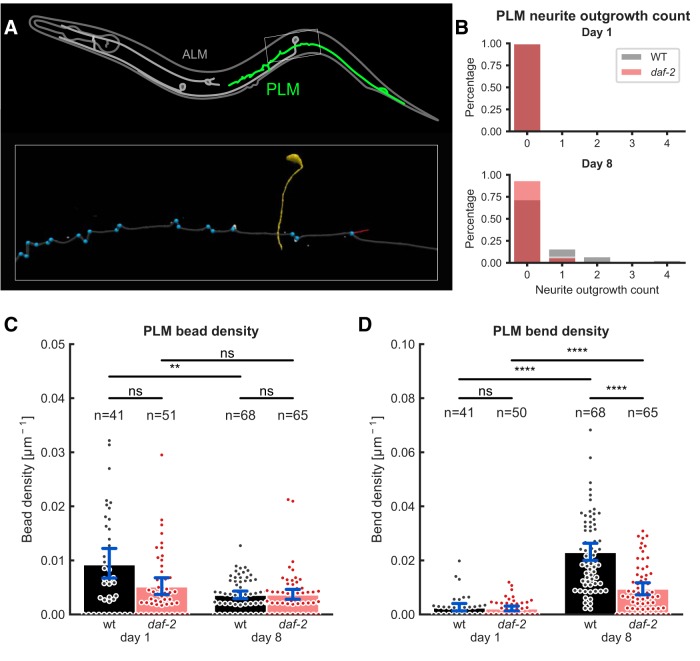
Age-related morphologic abnormalities of PLM neurons are less severe in long-lived *daf-2* mutants. ***A***, Overview of morphologic features quantified in PLM neurons. Inset shows enlarged rectangular region with quantifications indicated (neurite-outgrowth count and length, red; sharp bends, blue; beads not shown). Yellow structure is the crossing PVM neuron. ***B***, Neurite outgrowth counts at days 1 and 8 of adulthood for WT and *daf-2(e1370)* mutants. WT animals showed a significant increase in the number of neurite outgrowths between day 1 and day 8 (*p* = 3.15e-04), and higher neurite outgrowth counts than *daf-2(e1370)* mutants at day 8 (*p* = 1.12e-03). For direct comparison of this data with previous studies, we replotted this data in percentage of PLM neurons with neurite outgrowths (Extended Data Fig. 3-1). ***C***, A significant decrease in bead density (bead count divided by the total length of the neurite) is found for WT animals (*p* = 8.25e-03), but not for *daf-2(e1370)* mutants comparing day 1 to day 8, respectively. However, no significant difference between genotypes at day 1 or at day 8 was found. Error bars are 95% bootstrapped confidence interval of the mean, *n* is the number of PLM neurons scored. ***D***, Both WT and *daf-2(e1370)* mutants showed the same density of sharp bends (bend count divided by the total length of the neurite) at day 1 of adulthood (*p* = 0.903), whereas at day 8 of adulthood, there is a significant increase in bend density (day 1 vs 8: WT, *p* = 5.87e-18 vs *daf-2*, *p* = 8.34e-06). However, *daf-2(e1370)* mutants seems to protect against age-related wrinkly appearance of neuronal processes (*daf-2* vs WT, *p* = 3.85e-07). This is in contrast to *daf-2(RNAi)* shown in Extended Data Figure 3-2. Error bars are 95% bootstrapped confidence interval of the mean, *n* is the number of PLM neurons scored. Data were collected in three independent trials and pooled for the analysis shown in this figure (***B***–***D***). See Extended Data Figure 3-3 for different angle threshold settings and Extended Data Figure 3-4 the histogram of angles and corresponding cumulative distributions. The waviness caused by osmotic shrinkage of *C. elegans* looks different to the age-related sharp bends (Extended Data Fig. 3-5). ns = *p* > 0.05, ** = *p* < 0.01, *** = *p* < 0.001, **** = *p* < 0.0001. *Figure Contributions:* Max Hess made all the figures.

10.1523/ENEURO.0014-19.2019.f1-3 Extended Data Figure 1-3 Two approaches for the quantification of sharp bends. ***A***, left panel, For every node (red) along the main branch, an angle (α) was calculated by linearly approximating upstream and downstream nodes in a window of 2 μm (red and green). Middle panel, Colors represent the angles calculated for every node as specified in the first panel. Blue corresponds to wider angles, orange to narrow angles. Right panel, Nodes with minimum angles were selected sequentially (red dots), double counting was avoided by non-maximum suppression. ***B***, Visualization of the main branch backbone (red) and B-spline approximations of with differing amounts of smoothing (s). Blue colors correspond to parts of low curvature, orange to high-curvature. Scale bar = 2 μm. Download Figure 1-3, TIF file.

**Figure 4.**
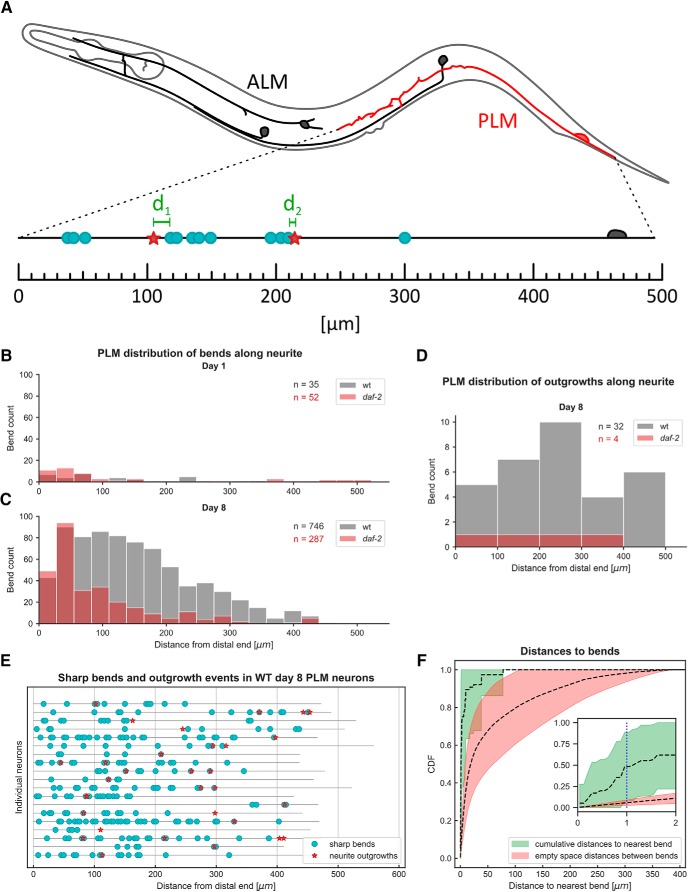
Correlation of sharp bends with neurite outgrowths of PLM neurons during aging. ***A***, Illustration depicting the procedure of determining the position of sharp bends (blue circles) and neurite outgrowths (red stars) along PLM neurons. The neuron was imagined to be stretch out along one dimension and the distances to the most distal point of the neuron was recorded. Furthermore, the distance to the nearest bend was recorded for every neurite outgrowth (green). ***B***, ***C***, Distribution of bends along the neurite for WT and *daf-2(e1370)* mutants at day 1 (***B***) and day 8 (***C***) shows distribution that bend occurrence is skewed toward the distal end of the neuron. ***C***, At day 8 of adulthood, WT neurons (gray bars) not only showed a higher count of sharp bends compared to *daf-2(e1370)* mutants (red bars), but their distribution was shifted toward the proximal region of the neuron (Kolmogorov–Smirnov two-sample *p* = 3.20e-125). n indicates the number of sharp bend events evaluated. ***D***, Distribution of outgrowth events along the neurite for WT and *daf-2(e1370)* mutants were not skewed toward the distal end. ***E***, Plot of the 19 WT day-8 PLM neurons that showed neurite branching. Black lines represent the main branch stretched out along one dimension, neurite outgrowths (red stars) and sharp bends (blue circles) are plotted at their position along the neurite illustrates that outgrowth events often occur in close proximity to bends. ***F***, CDFs of the distance to bends (confer A) and the ESD between bends. If outgrowths occurred independently of bends (meaning they were positioned randomly in the space between bends), one would expect the CDF to coincide with the empty space transformation. The course of the CDF compared to the ESD indicates that neurite outgrowths and sharp bends co-occur. As a statistical test, we assumed an interaction distance of 1 μm (inset, blue dotted line) and evaluated the individual CDFs and ESDs of the 19 WT day-8 neurons at that distance. A Wilcoxon signed-rank test showed that the percentage of outgrowth events that were 1 μm or closer to a sharp bend was significantly higher than one would expect if they were distributed randomly with respect to sharp bends (*p* = 2.90e-03). Data were collected in three independent trials and pooled for the analysis shown in this figure (***B***–***F***). *Figure Contributions:* Max Hess made all the figures.

10.1523/ENEURO.0014-19.2019.f1-4 Extended Data Figure 1-4 Heterogeneity of age-related morphological changes of observed in touch receptor neurons. Randomly chosen z-projections of WT *C. elegans* touch neurons at ages day 1 and day 8. Download Figure 1-4, TIF file.

Here, we use confocal imaging to obtain three-dimensional image-stacks of the shape of touch receptor neurons. We apply a neuron-tracing algorithm (all-path-pruning 2.0 (APP2); Xiao and Peng, 2013) to evaluate the resulting three-dimensional tree-representation. We further develop this analysis for *C. elegans* neuronal tracing and establish a workflow for a semi-automatic quantification pipeline. We examine the neuronal morphology of young versus aged (1 vs 8 d of adulthood) of WT and long-lived *C. elegans*. With our high-resolution analysis we demonstrate that (1) *C. elegans* show a decrease in ALM soma volume during aging, (2) longevity-promoting *daf-2* mutation significantly lowers the occurrence of neurite branching in PLM neurons, and (3) age-related occurrence of sharp bends along the sensory dendrites of PLM neurons are aggregated in distal parts of the neuron. Our quantitative analysis unveils a correlation between sharp neurite bends and neurite outgrowths. All data and code are made available to researchers at https://zenodo.org/record/2350066#.XBgUImhKiUl and https://github.com/HessMax/NeuronMorphologyQuantification. Our semi-automatic pipeline provides a framework to gain mechanistic insights in how neurons remain resilient in long-lived animals or into the etiology of these age-dependent morphologic changes.

## Materials and Methods

### Semi-automated pipeline outline

Step-wise overview of the pipeline to quantify the morphologic changes shown in Figure 1. Detailed description for each step is given in the accompanied paragraphs below.

#### Step 1: strains and handling

Prepare SK4005: *zdIs5* [P*mec-4*::GFP + *lin-15*(+)] transgenic strain that expresses GFP in the mechanosensory neurons (Clark and Chiu, 2003).

#### Step 2: image acquisition

Acquire confocal images of anterior lateral microtubule (ALM) and posterior lateral microtubule (PLM) touch receptor neurons (Extended Data Fig. 1-1*A*,*B*).

#### Step 3: pre-processing

First, stitch images of PLM neuron stacks (ImageJ; Preibisch et al., 2009) and then manually remove neuronal cell bodies or dendrites from other neurons than ALM or PLM (Extended Data Fig. 1-1*C*).

#### Step 4: neuron tracing

To generate three-dimensional tree representations, use all-path-pruning APP2 neuron tracing software (Xiao and Peng, 2013; Extended Data Fig. 1-1*D*).

#### Step 5: quantification of neuronal trees

To quantify morphologic changes from these neuronal trees, use our Python script (see corresponding section; Fig. 1; Extended Data Fig. 1-1*E*,*F*).

### Strains and handling

*C. elegans* strains were maintained on nematode growth media (NGM) plates with *Escherichia coli OP50-1* bacteria at 20°C as described by Brenner (1974). SK4005: *zdIs5* [*Pmec-4*::GFP + *lin-15*(+)] was used and is referred here as WT control. The *daf-2(e1370)* mutation was crossed into SK4005. Strains used in the experiments were maintained for at least two generations at 20°C. Synchronization was done by hypochlorite solution with direct transfer of the bleached eggs to NGM plates. *C. elegans* were grown to L4 at 20°C and transferred to 50 μM 5-ﬂuorodeoxyuridine (FUDR; Sigma-Aldrich F0503) plate to be maintained at 25°C (Teuscher et al., 2019). At day 1 of adulthood, ∼20 animals per condition were used for imaging, the rest were maintained to day-8 adulthood at 25°C for the second imaging session. For the experiments at 15°C, all processes are the same as above except that all *C. elegans* were always kept at 15°C. RNAi was performed as described in Ewald et al. (2017).

### Image acquisition

Images were taken with an upright confocal laser scanning microscope (CLSM) Leica SP8 with a 40×/1.00 oil objective. For excitation, a solid-state diode laser (OPSL 488) with a wavelength of 488 nm was used at an intensity of 1%. For detection, a hybrid detector (HyD) was set to the range of 493–556 nm with a gain of 10%. Images were recorded with a frequency of 700 Hz, 1.28× zoom and a resolution of 1024 × 1024 pixels leading to x and y pixel dimensions of 223 nm. Z-step size was set to 300 nm. Samples were mounted on microscope slides (76 × 26 mm) with a 4% agarose pad (Teuscher et al., 2019). First a 12-µl drop of 50 µM Levamisole (Sigma-Aldrich L0380000) was placed on the agarose pad. Twelve *C. elegans* were picked from the FUDR plates and deposited into the droplet. An eyelash pick was used to disperse the *C. elegans* in the droplet to ensure adequate spacing between them. Finally, a coverslip (18 × 18 mm) was placed on top. Depending on the orientation of the *C. elegans* on the microscope slide, either the left or right ALM and PLM neurons were imaged. If the left PLM neuron was imaged, the full soma of the PVM neuron was included in the image stacks to later be able to differentiate neurite outgrowths from the PVM crossing. *C. elegans* that were curled up or did not lie on the side were excluded from imaging. To reduce imaging time, only parts of the neurons were imaged (Extended Data Fig. 1-1). One stack was recorded including the ALM soma, and two stacks were recorded covering the distal part of the PLM neuron (Extended Data Fig. 1-1*A*,*B*). Using this imaging scheme, an average of five to six *C. elegans* were imaged per hour. Images were labeled STRAIN_SERIES_AGE_NEURON# and are available at https://zenodo.org/record/2350066#.XbgUImhKiUl.

### Quantification of neuronal morphology

#### Pre-processing

PLM image stacks were stitched (Extended Data Fig. 1-1*B*) using the ImageJ “pairwise-stitching” plug-in Preibisch et al. (2009). In ALM images, soma of AVM neurons that were in the imaged volume were removed manually using the “flood-fill tool” to ensure proper ALM-soma detection of the neuron tracing algorithm. In PLM stacks, processes from other neurons projecting into the image volume were manually removed from the images since those might otherwise interfere with the automatic initialization of the neuron tracking. On all acquired image-stacks, a Gaussian blur with sigma (0.7, 0.7, 0.52) was applied to smooth out any imaging noise and facilitate neuron tracing.

#### Neuron tracing

Neuron tracing was performed using the APP2 neuron tracing algorithm (Xiao and Peng, 2013) distributed in Vaa3D software package. APP2 algorithm was used in the command line mode via a shell script for batch processing. For the ALM neurons, APP2 algorithm was run with its soma detection feature enabled. For PLM neurons a root node was generated at the brightest location along the faces of the image volume (i.e., the location where the PLM process intercepts the outer boundaries of the imaging field-of-view), and saved as a .marker file to input to the APP2 algorithm. The algorithm settings were generally kept at default values, but re-sampling which was turned off and the gaps flag (allowing for small gaps) was turned on. Output of the neuron tracing algorithm were trees in the .swc file format which were stored for further processing.

#### Quantification of neuronal trees

We generated a semi-automated quantification pipeline to quantify the morphologic changes in aging *C. elegans* touch neurons. It includes measuring length and number of soma- and neurite-outgrowths, quantification of soma-volume and the densities of beads and sharp bends on individual neurites. The code is made available on https://github.com/HessMax/NeuronMorphologyQuantification and is provided in the Extended Data 1.

Extended Data File 1. Code and instruction for the neuronal quantification analysis. The zip file includes the python code files for batch processing, beads, classify, clean up, kink positions, soma volume, utility, and waviness. In addition, a swc example image, readme, requirements, and license text files are also included. *Figure Contributions*: Max Hess acquired confocal images. Max Hess, Alvaro Gomariz, Orcun Goksel, and Collin Ewald devised the image analysis pipeline, which was implemented by Max Hess and Alvaro Gomariz. Max Hess and Collin Ewald wrote the legends in consultation with the other authors. Download Extended Data F, ZIP file.

#### Classification of branches and length measurements

The output tree of the neuron tracing APP2 algorithm was used to classify the nodes into seven categories: mainbranch, soma, neuriteoutgrowth, somaoutgrowth, pvm, vnc_connection, and blob_artifact (Fig. 1).

For ALM neurons, all the nodes adjacent to the root node that were above an empirically determined radius threshold were classiﬁed as soma nodes. Next, the distances from every endpoint to the soma was calculated by adding up all the individual Euclidean distances between consecutive nodes along the neuron. The nodes of the longest branch were classiﬁed as the main branch. Side-branches ending in a soma node were classiﬁed automatically as soma outgrowths, whereas the side-branches ending in a main-branch node were classiﬁed as neurite branches.

For PLM neurons, the ﬁrst step was also to calculate the distances from every endpoint to the root. Again, the longest branch was deﬁned to be the main branch. As PLML neurons cross the proximal part of the PVM neuron very closely, about half of the traced PLM neurons incorrectly contained a part of the PVM neuron as a side-branch. These incorrect branches were ﬁrst classiﬁed by selecting a side-branch with nodes above the soma radius threshold (effectively looking for the PVM soma in the side-branches). A region of 4 µm around this side-branch’s branching point on the main-branch was selected to be “PVM branching nodes” and all the side-branches terminating in those nodes were classiﬁed as PVM-neurons (Extended Data Fig. 1-1*E*). Finally, all the other side-branches were classiﬁed as neurite-branches and their lengths and locations along the main branch were recorded. As PLM neurons have one (sometimes even two) connections to the PVM neuron, which our algorithm could not differentiate from neurite-branches, we manually annotated those branches in the raw neuron-trees using the open-source neuron-tree editing and visualization software “neuTube” (www.neutracing.com; Feng et al., 2015).

The resulting automatically estimated outgrowths are demonstrated to have good correlation (*R* = 0.967) with the manually annotated lengths for nine samples (Extended Data Fig. 1-2*A*,*B*).

#### Soma volume

ALM soma volumes were segmented using the ITK Insight Toolkit for image segmentation and registration (www.itk.org) with the SimpleITK.ConfidenceConnected region growing algorithm followed by a morphologic opening operation to clean up the segmentation, whereby the soma-node detected by APP2 was used as a seed. Soma volumes were then estimated by adding up the volumes of the segmented voxels. A comparison of automatically detected soma volumes to a manually annotated ground truth showed a strong correlation (*R* = 0.962 for 11 samples) between manual and automatic measurements (Extended Data Fig. 1-2*C*,*D*).

#### Beads and blebs

The radius information from the neuron-tracing output of the main branch was used to detect larger masses (so-called “blobs” in computer vision) along the neuronal process. Such blobs indicate beads, which are focal enlargements along neuronal processes (Pan et al., 2011), or blebs, which are triangular-shaped protrusions along neurite processes (Pan et al., 2011). To account for variations in branch thickness along the process, the local thickness was calculated in 8-μm windows and subtracted from the node radii. The nodes with maximum radii were sequentially counted as a blob if they were more than two standard deviations thicker than the mean process. The bead/bleb density was then calculated by dividing the blob count of a process by its length. This approach was verified visually and showed confirmative results.

#### Sharp bends (kinks) along the main branch

For every node of the main branch, an angle was calculated by ﬁrst selecting neurite nodes within an accumulated distance of 2 µm along the branch. Having fitted two lines separately to upstream and downstream nodes, the angle between these two lines were computed from the cross-product (Extended Data Fig. 1-3*A*). From all such potential points, those with an angle smaller than a threshold of 155° are marked as a sharp bend (Extended Data Fig. 1-3*A*). To prevent double counting of bends, the bends were selected sequentially starting with the sharpest, and after every selection of a bend, by marking the nodes within a local-neighborhood of 4 µm to exclude them from future detection (i.e., so-called non-maximum suppression). Alternatively, a second approach was also implemented and tested, where the whole main-branch is approximated with a B-spline (Extended Data Fig. 1-3*B*), where the local curvature of the spline was used to assess neurite bending. This spline-fitting approach turned out to be less robust to noise based on evaluations against manual annotations, thus it was eventually abandoned. The above method automatically detected sharp bends successfully, based on an evaluation with manual counts from visual inspection as ground truth. Despite the large variance of individual observers’ assessment on what qualifies as a “sharp bend” in neurites, our automatic method is very close to the mean of five individual assessors for 12 neurite samples with on-average 13.75 bends (Extended Data Fig. 1-2).

#### Association of sharp bends and neurite outgrowths

Visual inspection of the images leads to the impression that neurite outgrowth events often occur at locations where the sensory dendrite shows a sharp bend. To test this hypothesis, we employed point processes, a validated framework of spatial statistics, to study spatial trends in a similar way as other quantitative microscopy studies (Gomariz et al., 2018). Accordingly, we studied the potential interactions between sharp bends and neurite outgrowths by first calculating the empty space distance (ESD) between sharp bends, and then comparing this to the minimum distance of every neurite outgrowth to the nearest bend. In this way, the ESD provides baseline information about how the sharp bends regulate the space for the outgrowths to distribute. Presenting the cumulative distribution function (CDF) of the distances between these outgrowths also allow for an easier interpretation of results as well as comparisons across different experimental settings.

### Software and data accessibility

The processing pipeline and the full dataset used in this work will be posted to public repositories on acceptance of the paper. The code is provided in the Extended Data 1. The code was tested on Windows 10, Linux, and Mac using Python 3.6.7.

## Results

### Acquisition of age-related morphologic changes of touch receptor neurons

Different age-associated morphologic changes are displayed more prominently in different neurons (Tank et al., 2011; Toth et al., 2012), being of particular interest are the ALM and PLM touch receptor neurons (Fig. 1). In previous studies (Pan et al., 2011; Tank et al., 2011; Toth et al., 2012), different neuronal features of *C. elegans* were described by visual inspection, including the number of bends/kinks of the main sensory dendrite (also called wavy processes), the number of beads along the main sensory dendrite, and the number of novel outgrowth of processes either from the main sensory dendrite (also called ectopic branches or neurite sprouting) or from the soma. By analyzing three-dimensional tree representations of neurons generated from confocal image stacks (Extended Data Fig. 1-1), we validated that our semi-automated pipeline quantification is similar to the previous manually obtained features listed above (Extended Data Fig. 1-2). Furthermore, we quantified additional morphologic features including the length of the new processes sprouting from the soma or the main sensory dendrite, the soma volume, and the number and location of beads as well as sharp bends (kinks; Extended Data Fig. 1-3) forming along the main sensory dendrite (Fig. 1). The heterogeneity of these age-related morphologic changes is illustrated in Extended Data Figure 1-4. Thus, we capture a more complete spectrum of potentially age-related morphologic changes of these neurons.

### Age-associated morphologic aggravations of ALM neuron are decreased in long-lived *C. elegans*

The ALM neurons show novel soma outgrowths during aging (Pan et al., 2011; Tank et al., 2011; Toth et al., 2012). Cytoskeleton outgrowths of microtubules are found in these ALM soma protrusions (Pan et al., 2011). With our image analysis pipeline, we detected the ALM soma outgrowths as illustrated in Figure 2*A*. The method was employed for quantifying the outgrowths in WT and long-lived *daf-2(e1370)* mutant *C. elegans*, first when they were young (day 1 of adulthood) and then again when they were old (day 8 of adulthood). In agreement with the number of ALM soma outgrowths from earlier studies (Toth et al., 2012; Extended Data Fig. 2-1*A*), long-lived *daf-2(e1370)* mutants showed a reduced number of novel outgrowths at day 8 of adulthood cultured at 25°C (Fig. 2*B*). In addition to previous studies, we found that the lengths of individual ALM soma outgrowth processes increased with age in WT, but no significant difference was found in long-lived *daf-2(e1370)* mutants (Fig. 2*C*). A second novel observation was that ALM soma volume decreases with age (Fig. 2*D*). Although the body size of *daf-2(e1370)* mutants at 25°C is smaller than WT (Ewald et al., 2015), the ALM soma volume of *daf-2(e1370)* mutants at 25°C is bigger than WT (Fig. 2*D*). Surprisingly, the ALM soma volume of *daf-2(e1370)* mutants also shrinks with age to the same extent, with ∼30% soma volume decrease from day 1 adulthood similarly to WT (Fig. 2*D*). This new finding led to the hypothesis that the decrease in soma volume might be due to cytoskeleton restructuring that occurs in the context of novel process sprouting from the soma. Correlation analysis of the variables “soma volume” and “total soma outgrowth length” (i.e., adding up the lengths of all soma outgrowths) at day 8 of adulthood did not show any correlation for WT animals (Fig. 2*E*) and only a slight correlation for *daf-2(e1370)* mutants (Fig. 2*F*). Based on these data, we discard the hypothesis that new processes sprouting from the soma can account for the decrease in soma volume. Interestingly, during very old age (day 21 of adulthood at 15°C), *daf-2(e1370)* mutants still have increased ALM soma volume compared to WT, but the age-related shrinkage observed in both conditions at 25°C was not seen at 15°C (Extended Data Fig. 2-1*B*). Although, in 21-days-old *daf-2(e1370)* mutants at 15°C, the number of ALM soma outgrowths were not significantly decreased (Extended Data Fig. 2-1*C*) compared to WT, the lengths of individual soma outgrowths were slightly longer in the long-lived *daf-2(e1370)* mutants (Extended Data Fig. 2-1*D*). This suggests that long-lived *daf-2(e1370)* mutants assures cellular morphology by having bigger soma volumes in addition to preventing novel outgrowths during aging.

10.1523/ENEURO.0014-19.2019.f2-1 Extended Data Figure 2-1 Comparison of ALM morphological changes of three-week-old *C. elegans* maintained at lower temperature. ***A***, Percentage of ALM neurons with soma outgrowths of WT and long-lived *daf-2(e1370)* at day 1 and day 8 of adulthood at 25°C. Same data as in Figure 2*B* plotted as percentage to make it comparable to previous studies. ***B***, ALM soma volume of animals kept at 15°C until day 21 of adulthood showed the significant difference in soma-volume between WT and long-lived *daf-2(e1370)* mutants. ***C***, WT and long-lived *daf-2(e1370)* mutants showed no significant difference in the number of soma outgrowths at day 21 of adulthood at 15°C. ***D***, Contrary to our results at day 8 of adulthood at 25°C, the lengths of soma outgrowths are longer in *daf-2(e1370)* mutants compared to WT at 15°C at day 21 of adulthood. Download Figure 2-1, EPS file.

### Some but not all age-associated morphologic aggravations of PLM neuron are decreased in long-lived *C. elegans*

In contrast to the predominant soma outgrowths of ALM neurons, the PLM neurons show novel neurite outgrowths along the main sensory dendrite (Fig. 3*A*,*B*). A significant increase in the number of outgrowth events along the main branch could be observed for WT animals when comparing young (day 1) and 8-days-old adults (Fig. 3*B*). We report a slightly higher number of branching events compared to previous studies (Toth et al., 2012), likely because our method allows the detection of small neurite outgrowths (Extended Data Fig. 3-1*A*). Furthermore, despite the low occurrence rate of neurite branching events, our method registered a significantly lower number of branching events in long-lived mutants compared to WT (Fig. 3*B*; Extended Data Fig. 3-1*A*). This result is in accordance with a previous study, where manual inspection of the number of PLM neurite outgrowths showed a lower count in *daf-2(e1370)* mutants, however, did not reach statistical significance (Toth et al., 2012). Interestingly, in very old adults at 15°C, there is no difference in neurite outgrowth counts between *daf-2(e1370)* and WT (Extended Data Fig. 3-1*B*), suggesting that in long-lived *daf-2(e1370)* mutants this process is slowed down but not entirely prevented. We also investigated whether in 8-days-old WT and long-lived *daf-2(e1370)* mutants the PLM neurite outgrowth lengths would differ, but this was not the case (Extended Data Fig. 3-1*C*). These findings suggest that *daf-2* might protect against the incidences of neurite outgrowths earlier during the adult lifespan. However, reducing *daf-2* by RNA interference does not protect against these age-dependent changes (Extended Data Fig. 3-2), suggesting an alternative hypothesis about dauer-dependent mechanisms (see Discussion).

10.1523/ENEURO.0014-19.2019.f3-1 Extended Data Figure 3-1 Comparison of PLM morphological changes of three-week-old *C. elegans* maintained at lower temperature. ***A***, Percentage of PLM neurons with neurite outgrowths of WT and long-lived *daf-2(e1370)* at day 1 and day 8 of adulthood at 25°C. Same data as in Figure 3*B* plotted as percentage to make it comparable to previous studies. ***B***, Measurements of individual neurite outgrowths lengths observed in WT and *daf-2* day-8 animals raised at 25°C does not show a significant difference due to the overall low number of observations of neurite branches (especially in *daf-2* mutants). ***C***, Neurite outgrowth count of WT and *daf-2* animals raised at 25°C for 21 d does not show a significant difference between conditions (*p* = 0.837; WT *n* = 17, *daf-2 n* = 16). ***D***, Bend density of WT and *daf-2* animals raised at 25°C for 21 d does not show a significant difference between conditions (*p* = 0.911). Download Figure 3-1, TIF file.

10.1523/ENEURO.0014-19.2019.f3-2 Extended Data Figure 3-2 Reducing insulin/IGF-1 signaling via *daf-2* knock-down does not protect against age-dependent neuronal morphological abnormalities. ***A***, Both empty vector control and *daf-2* RNAi knock-down showed a significant increase in soma outgrowth counts between day 4 and day 13 of adulthood (empty vector control, *p* = 9.48e-03; *daf-2* RNAi, *p* = 1.29e-03) but *daf-2* RNAi knock-down did not have a significant effect (day 4, *p* = 0.684; day 13, *p* = 0.667). ***B***, Only *daf-2* RNAi knock-down showed a significant increase in neurite-outgrowth counts between day 4 and day 13 of adulthood (empty vector control, *p* = 7.56e-02; *daf-2* RNAi, *p* = 1.71e-03), and showed higher outgrowth counts at day 13 (*p* = 2.78e-02). ***C***, Both empty vector control and *daf-2* RNAi knock-down showed a significant increase in bend counts between day 4 and day 13 of adulthood (empty vector control, *p* = 2.37e-08; *daf-2* RNAi, *p* = 1.44e-08) but *daf-2* RNAi knock-down did not have a significant effect (day 4, *p* = 0.102; day 13, *p* = 0.089). Download Figure 3-2, EPS file.

10.1523/ENEURO.0014-19.2019.f3-3 Extended Data Figure 3-3 Robustness of PLM bend densities across different angles threshold settings. Using the data from Figure 3*D* with different angle thresholds, ranging from 135° to 165°, are quantified and show similar results of bend density across genotype and age. As expected, increasing the threshold leads to higher bend densities, but the differences between conditions are insensitive to changes in threshold angle settings. Dots show individual measurements and blue error bars are bootstrapped 95% confidence interval. Please note, the differently scaled axes between the rows. Download Figure 3-3, TIF file.

A second morphologic feature of PLM neurons are the bead-like structures along the dendrite. In accordance with existing literature (Pan et al., 2011; Toth et al., 2012), the bead count decreases between day 1 and day 8 of adulthood for WT (Fig. 3*C*). As only large beads could be detected by our approach, our counts were generally lower than those reported in the literature. Interestingly long-lived *daf-2(e1370)* mutants showed significantly lower bead density at day 1 compared to WT, but no difference was observed at day 8 of adulthood (Fig. 3*C*).

A third morphologic feature of the main sensory dendrites of PLM neurons is their wrinkly or wavy appearance with age (Pan et al., 2011; Toth et al., 2012). To quantify this wavy appearance, we calculated an angle in every node along the main process of our neuron model and counted nodes above a threshold as bends (see Materials and Methods, Sharp bends). A strong increase in the number of sharp bends per unit length (i.e., bend density) is observed during aging for both WT and *daf-2(e1370)* mutants (Fig. 3*D*). Strikingly, WT animals showed more than twice the bend density at day 8 of adulthood compared to *daf-2(e1370)* mutants (Fig. 3*D*); a finding that is insensitive to different angle threshold settings between 135° and 165° (Extended Data Figs. 3-3, 3-4). In older *daf-2(e1370)* mutants (day 21 of adulthood at 15°C) the density of sharp bends was not significantly different between conditions (Extended Data Fig. 3-1*D*), an observation consistent with the hypothesis that *daf-2* might be either protective early during the adult lifespan or act via dauer-dependent mechanisms (see Discussion). Since *C. elegans* shrink and become wrinkled during aging probably due to a decrease of internal pressure (Wolkow et al., 2017), we hypothesized that the increased number of bends during aging might be due to an overall compression of body length. To test this hypothesis, we placed 3-days-old *C. elegans* in 1.7 M salt right before imaging, which led to body shrinkage and also a wavy appearance of PLM neurons (Extended Data Fig. 3-5). However, this osmotic-induced waviness was visually different (rounder curves) compared to the characteristic sharp bends of PLM neurons during aging (Extended Data Fig. 3-5). Our osmotic-induced waviness looked similar to the waviness induced by increasing mechanical tension in young animals (Krieg et al., 2017). We conclude that the age-related sharp bends are less likely to be formed by simple loss of internal pressure or shrinkage of *C. elegans*.

### The probability of a sharp bend occurring rises with increasing distance to the PLM soma

Our method allows not only to count the number of bends, but also to understand their distribution along the neuron. First, we assessed the spatial correlation between the bends and the distal end of the neuron by measuring their relative distances (Fig. 4*A*). We could not use the PLM soma as a reference point as we only imaged the distal ends of PLM neurons (Extended Data Fig. 1-1*A*). Evaluation of the distribution of sharp bends along the main sensory dendrite showed a higher number of bends at more distal parts of the neuron and lower number at proximal parts (Fig. 4*B*,*C*). Strikingly, WT animals did not only show a higher number of sharp bends, but a shift in the distribution such bends toward the proximal part of the neuron (Fig. 4*C*). This suggests that reducing insulin/IGF-1 signaling with *daf-2(e1370)* mutation is protective preferentially toward the proximal part of the neurite closer to the soma.

### Positive correlation between neurite bending and novel neurite outgrowths

Next, we studied the distribution of PLM neurite outgrowths by applying the same statistical analysis technique of distances as in the previous section (Fig. 4*A*). Neurite outgrowths in our samples did not show a clear distribution along the PLM dendrites (Fig. 4*D*). However, a visual inspection revealed that the PLM neurite outgrowths often sprouted from a sharp bend (Fig. 4*E*). To assess this observation, we compared the CDF of the ESD between sharp bends and the CDF of the measured distances from outgrowths to nearest bend averaged over all day-8 WT PLM neurons. We found that the distances from the outgrowth location to the nearest bends are smaller than what would be observed as random occurrences (Fig. 4*F*). Furthermore, we assumed an interaction distance of 1 µm and performed a Wilcoxon signed-rank test between the two CDFs at that distance (Fig. 4*F*). This suggested that neurite bends are located preferentially near outgrowths.

## Discussion

In this study, we combined confocal imaging with neuron-tracing algorithms to reveal the subtle morphologic changes of neurons during aging. In addition to visual inspection of aged neurons, our automated image analysis methods revealed novel age-related morphologic changes, such as size of neurite outgrowths, soma volume, and location of the morphologic abnormalities. The advantage of obtaining exact measurements allows for high confidence temporal and genotypic comparisons and for studying correlations and novel hypotheses. This becomes particularly important for genetic screens to identify and validate modifiers of neurite morphology. In addition, the use of validated image analysis and statistical methods in the form of a semi-automatic quantitative microscopy pipeline is beneficial not only for the results presented herein, but also as a standardized benchmark for future studies.

Visual inspection of PLM neurite outgrowths during aging showed that the counts were not significantly decreased in long-lived animals (Toth et al., 2012). Our quantitative approach, however, allowed the detection of a significant difference between WT and long-lived *C. elegans*. Furthermore, we showed that not all interventions that lead to longevity prevent age-related neuronal changes. For instance, the genetic model of dietary restriction (*eat-2* mutants) or knocking down *daf-2* by RNAi (Tank et al., 2011) both did not necessarily prevent age-related neuronal changes as we quantified them. Having repeated the *daf-2* RNAi knock-down experiment by visual inspection, we did not observe any differences in the occurrence of morphologic abnormalities of long-lived *daf-2(RNAi)* animals compared to control animals (Extended Data Fig. 3-2). By contrast, using the *daf-2(e1370)* mutant at 25°C as in previous studies (Tank et al., 2011; Toth et al., 2012) and our study showed clear improvements of these age-dependent neuronal deficits. Since neurons are insensitive to RNAi (Calixto et al., 2010), one hypothesis would be that *daf-2* needs to be reduced in neurons to cell-autonomously slow the occurrence of these age-related morphologic changes in the touch receptor neurons. Inconsistent with this hypothesis is the finding that long-lived *daf-2(e1370)* mutants at 15°C showed no significant protection against these age-related morphologic abnormalities (Extended Data Fig. 3-1). To reconcile these contrasting findings, we propose an alternative hypothesis. Reducing *daf-2*/insulin/IGF-1 receptor signaling can increase lifespan via dauer-dependent and dauer-independent mechanisms (Ewald et al., 2015). Activation of dauer-dependent mechanisms induced in *daf-2(e1370)* mutants at 25°C results in substantial remodeling of body composition, changes in the electrical connectome (Bhattacharya et al., 2019), and behavioral changes, whereas no behavioral nor morphologic remodeling is observed under long-lived dauer-independent conditions (for review, see Ewald et al., 2018), such as *daf-2* RNAi at any temperature or *daf-2(e1370)* mutants at 15°C (Ewald et al., 2015). Hence, we hypothesize that the different effects on neuronal morphologic protection of *daf-2(e1370)* versus *daf-2(RNAi)* might be due to improper activation of dauer-dependent mechanisms. Our results are consistent with this alternative dauer-dependent mechanism of reduced insulin/IGF-1 receptor signaling protects against such age-related morphologic changes. This might also explain why not all long-lived interventions (such as dietary restriction via *eat-2* mutation) result in slowing down the occurrences of these progressively accumulating abnormalities. In the future, additional experiments might identify the molecular underpinning of how dauer-dependent mechanisms prevent or remodel these age-related morphologic changes.

Our observation of the soma volume decreases with age, whereas soma outgrowth lengths increase with age, led to the hypothesis that cytoskeletal material from the soma might be used for the soma outgrowths. In line with this idea would be the observation of substantial cytoskeletal remodeling of the soma as well as these novel soma outgrowths containing acetylated microtubules (Pan et al., 2011). However, we have found no correlation between increased soma outgrowth length with decreased soma volume. A limitation could be that we assumed the recycling of cytoskeleton building blocks. As observed with the association of the mitochondria next to the neurite outgrowths, the soma outgrowths might be energy-driven and novel synthesis of microtubules. Another tantalizing hypothesis we had, but did not test, was that the loss of soma volume might be due to a recently descripted exopher clearing mechanism. *C. elegans* neurons extrude membrane-surrounded exophers filled with aggregated proteins and damaged mitochondria (Melentijevic et al., 2017). These exophers can become as large as 4 μm in size, similar to the typical size of neuronal soma (Melentijevic et al., 2017). Especially, ALM neurons frequently produce these exophers (Melentijevic et al., 2017). We found that the ALM soma volume was larger in long-lived animals that have been shown to prevent protein aggregation and keep mitochondria intact.

During normal aging, no correlation between severity of morphologic changes and defective touch response has been found (Toth et al., 2012). However, touch sensitivity declines with aging (Pan et al., 2011; Tank et al., 2011). Furthermore, during normal aging neurons do not die (Herndon et al., 2002) and the nuclei of the six touch receptor neurons stay intact (Pan et al., 2011), suggesting that a functional decline of aged touch receptor neurons occurs in the absence of apoptosis or necrosis. Interestingly, a correlation between decreased age-dependent mobility and extra neurite sprouting was found earlier (Tank et al., 2011). Both the progression of age-dependent decline in mobility and an increase in morphologic changes in touch receptor neurons are slowed down in long-lived animals or are accelerated in short-lived animals (Pan et al., 2011; Tank et al., 2011; Toth et al., 2012). Longitudinal studies of these morphologic changes showed that neurite processes can grow out and retract within days (Pan et al., 2011), suggesting the transient and dynamic nature of these abnormalities.

In line with previous reports (Herndon et al., 2002; Pan et al., 2011; Toth et al., 2012), we did not observe any neurodegeneration (i.e., neuronal loss) during normal aging in the 514 neurons we had imaged. For instance, Toth and colleagues examined over 1100 touch receptor neurons and found no incidences of neurite breakage or neuronal apoptosis or necrosis (Toth et al., 2012). Thus, during normal *C. elegans* aging, neurons are not degenerated but rather undergo morphologic alterations.

The picture emerging from this work is that some morphologic changes might be connected, such as sharp bends correlating with neurite outgrowths. Although our analysis does not allow us to infer a causal direction between the two, it is tempting to speculate that sharp bends proceeds neurite outgrowths. However, this raises the question of what stimulates neurite outgrowth in old animals? We found that the PLM dendrite preferentially shows these age-dependent sharp bends at the distal part of the dendrite. Based on previous studies, we propose the following hypothetical model of how these neurite outgrowths might be stimulated. (1) Previously, genetic mutants that promote loss of neuronal attachments to either the extracellular matrix or hypodermis showed age-dependent increase in bending and neurite outgrowths (Pan et al., 2011). This suggests that during normal aging the neuronal attachment weakens. (2) Loss of neuronal-specific microtubule-associated proteins with actin-spectrin may lead to sharp bends of dendrites and even to super-coils with increased mechanical tension in young animals (Krieg et al., 2017). This suggest that internal neuronal structures, such as microtubule and actin-spectrin networks, are important to maintain dendritic shape. During aging, it might be harder for neurons to deliver microtubule and actin-spectrin building blocks from the soma to the distal part of the dendrites. (3) Mitochondria have been associated strongly with the sharp bends that show neurite outgrowths (Toth et al., 2012). These mitochondria might be simply stuck at these sharp bending sites, but might also provide energy to stimulate the neurite sprouting. Together with our correlation of outgrowths occurring near bending sites, we speculate that the weakening of neuronal attachment and progressive decline in cytoskeletal integrity during aging may be leading to bending of the neurites where mitochondria start to accumulate to stimulate novel neurite outgrowths. For future research, it will be exciting to experimentally assess this model. Our method will aid future research to quantify and analyze morphologic changes of *C. elegans* neurons during aging. Understanding the molecular mechanisms that underlie the aging process of *C. elegans* neurons may also shed light on analogues processes in mammals and on the aging human brain.

10.1523/ENEURO.0014-19.2019.f3-4 Extended Data Figure 3-4 Histogram and CDFs across angles. Using data from Figure 3*D*, histogram and cumulative distribution of angles are plotted. ***A***, Histogram showing angle measurements of all PLM main branch nodes for WT and *daf-2(e1370)* mutants at days 1 and 8 of adulthood. ***B***, Corresponding cumulative distributions of all angles for WT and *daf-2(e1370)* mutants at days 1 and 8 of adulthood. Download Figure 3-4, TIF file.

10.1523/ENEURO.0014-19.2019.f3-5 Extended Data Figure 3-5 Osmotic shrinkage of *C. elegans* results in bending of PLM neurons that look different than the age-related sharp bends. ***A***, Fluorescent image of a typical PLM neuron at day 4 of adulthood shows beads along the process but no sharp bends. ***B***, Fluorescent image of a typical PLM neuron at day 13 of adulthood shows a couple of sharp bends along the process. ***C***, Fluorescent image of a typical PLM neuron at day 3 of adulthood that was imaged upon placing the *C. elegans* in a drop of 1.7 M NaCl to induce osmotic pressure. The induced wrinkles appear different (rounder curvature as observed when neurons supercoil; Krieg et al., 2017; see Discussion) than the age-related morphology. For ***A–C***, insets are 3× zoom of dashed rectangles. Scale bars = 20 μm. Download Figure 3-5, TIF file.

## Synthesis

Reviewing Editor: Jörg Schulz, RWTH Aachen University Decisions are customarily a result of the Reviewing Editor and the peer reviewers coming together and discussing their recommendations until a consensus is reached. When revisions are invited, a fact-based synthesis statement explaining their decision and outlining what is needed to prepare a revision will be listed below. The following reviewer(s) agreed to reveal their identity: Itzhak Mano.

In this article the authors use C elegans as a model organism to quantify and describe age-induced morphological changes in a subset of the mechanosensory neurons. The authors focus on the neurons responsible for the sense of touch, due to the wide data accessibility in the community allowing them to compare their results with existing knowledge. In addition, they contribute to the quantitative description of the age-induced defect by supplying a high-resolution quantification of kinks and aberration that was unmatched before, proposing a new standard in the field. The derived knowledge from their comparison between wildtype ageing and the longevity mutation daf-2 is incremental, but might help researchers in the field to narrow down their experimental design and microscopy. Most importantly, the authors present an semi-automated pipeline, composed of image processing, neurite tracing and data collection, taking advantage of previously presented software packages, namely APP and ITK. The data collection stream is set up in open source computer code in Python to \ extract the relevant parameter, such as soma volume, kink density etc. Such a program is indeed a milestone for the community and was long been waiting for such a tool as evidenced by the huge amount of data sets available and published. This is very exciting. Despite the timely presentation of the novel pipeline. However, the versatility of the code significantly lacks behind of the expectations is creates. The code should become more versatile and not a single user source. For how it stands now, the code is unlikely to be applied by a large community.

The first reviewer states: The authors should decide, whether they want to communicate a resource or research paper, but for now, neither is really clear, but can be fixed in a revision.

If the authors want to present a resource that is widely applicable, I suggest a step by step instructions. Another drawback is that the algorithm relies on neuron tracing from a different software and I the procedure to use this needs to be explained as detailed as the original Python routine presented in here.

The code is poorly annotated and I wish the authors had taken their time to more explicitly describe the operations behind each line and the input/output data structures. Not everyone in the C elegans ageing field is familiar and fond of running code from scratch.

Some packages are not compatible with other platforms than windows. E.g. there is no straightforward workaround to winsound, except commenting the lines where it appears or directly addressing the speakers to make a beep. Perhaps the authors can include a versatile solution if sounds are necessary.

The code run perfectly on the presented sample data. However, are there alternatives to APP preprocessing and to extract the swc files? Also, the data structure seems to be hardcoded but could be prompted to provide more flexibility. Again, opening the code up for wide use also requires to handle different data structure, or a very detailed description of how the data needs to be presented.

What do the input parameters mean? A more detailed description is necessary.

The authors introduce the quantification of the bead density, but mention that they will not use it for further comparison, because it is not an age-related phenotype. However, beading and blebbing is a common signature of nonfit and stresses neurons, so I strongly recommend to add this quantification in their data pipeline.

page 11: The classification of sharp bends is very qualitative. Can the author extract all angles and plot the distribution.

Animal immobilized if levamisole hypercontract, which changes neuron morphologies. The authors make a similar statement on page 18. Does the kink angle change in animals immobilized in ivermectin?

page 16: The authors use daf-2 RNAi to investigate a neuronal phenotype. Does the absence of a phenotype upon treatment not relate to the RNAi sensitivity of neurons?

Minor points:

etiology instead of aetiology

throughout: PVM instead of PMV

page 6 line 108: hypochlorite

page 14: fuller-spectrum > more complete

The second reviewer states: The paper presents an interesting and important technology for streamline analysis of visual data of neurite morphology in the worm. This technology is used here to analyze changes in the shape of neurites during normal aging, and in mutants where aging is slowed down. However, the technology has a much broader potential to be useful for a wide range of applications in the study of the morphology of the nematode nervous system (which is a critical factor in nervous system physiology).

The method presented seems valid, as it is reasonably able to recapitulate similar observations made previously by manual image analysis. The semi-automated analysis seems to have some shortcomings, e.g., where daf-2 effects, which in previous manual studies seemed very large, seem here to be smaller (though significant). Nonetheless, the wide applicability of this method to genetic screens and other studies of factors that affect the morphology of neuronal processes, suggests that this method is of significant interest to the neuroscience community.

could not open the extended data file. It seems that it includes computer code for the analysis program used in this study. As such it is likely to be useful for future users. However, even if it were available to me, I do not have the training to judge the appropriateness of the coding.

Minor points:

1) The manuscript suggests that it shows new evidence in several points. However, many of these points represent incremental advances to effects that have been described (at least to some degree) by previous studies (e.g., changes in ALM cell body morphology with aging, or that PLM morphology changes are reduced in aging mutants). A particularly interesting new observation is the one revealing that increased bending in neurite morphology happens primarily in the initial segment of the neurite. The manuscript can be better focused by more accurately differentiating between valid reconfirmation of previous observations (establishing the accuracy of this less labor-intensive analysis), and novel findings.

2) The automated system seems unable to detect previously observed changes in beading. Previously described age-associated defasciculation has not been addressed or discussed here at all. The manuscript can be improved by further discussing these aspects.

3) The discussion should be more focused on the issue at hand. Neurodegeneration models have not been studied here, so there is no benefit to speculating about their effect on the issue studied here. In contrast, genetic screens and analysis of modifiers of neurite morphology are an important application that has not been sufficiently addressed.

4) Fig 2 : “719 G-I, Classified PLM tree with main-branch (grey), neurite-outgrowth (red) crossing PMV neuron (yellow) and sharp bends (blue) in frontal (G) and top (I) view.” I guess the authors mean: “ Lateral (G) and Dorsal (I) view”.
