# Supplementary figures and images for: In-Vivo Quantitative Image Analysis of Age-Related Morphological Changes of C. elegans Neurons Reveals a Correlation between Neurite Bending and Novel Neurite Outgrowths
Source: eNeuro. 2019 Jul 8;6(4):ENEURO.0014-19.2019. doi: 10.1523/ENEURO.0014-19.2019 (PMC6620389; doi:10.1523/ENEURO.0014-19.2019)

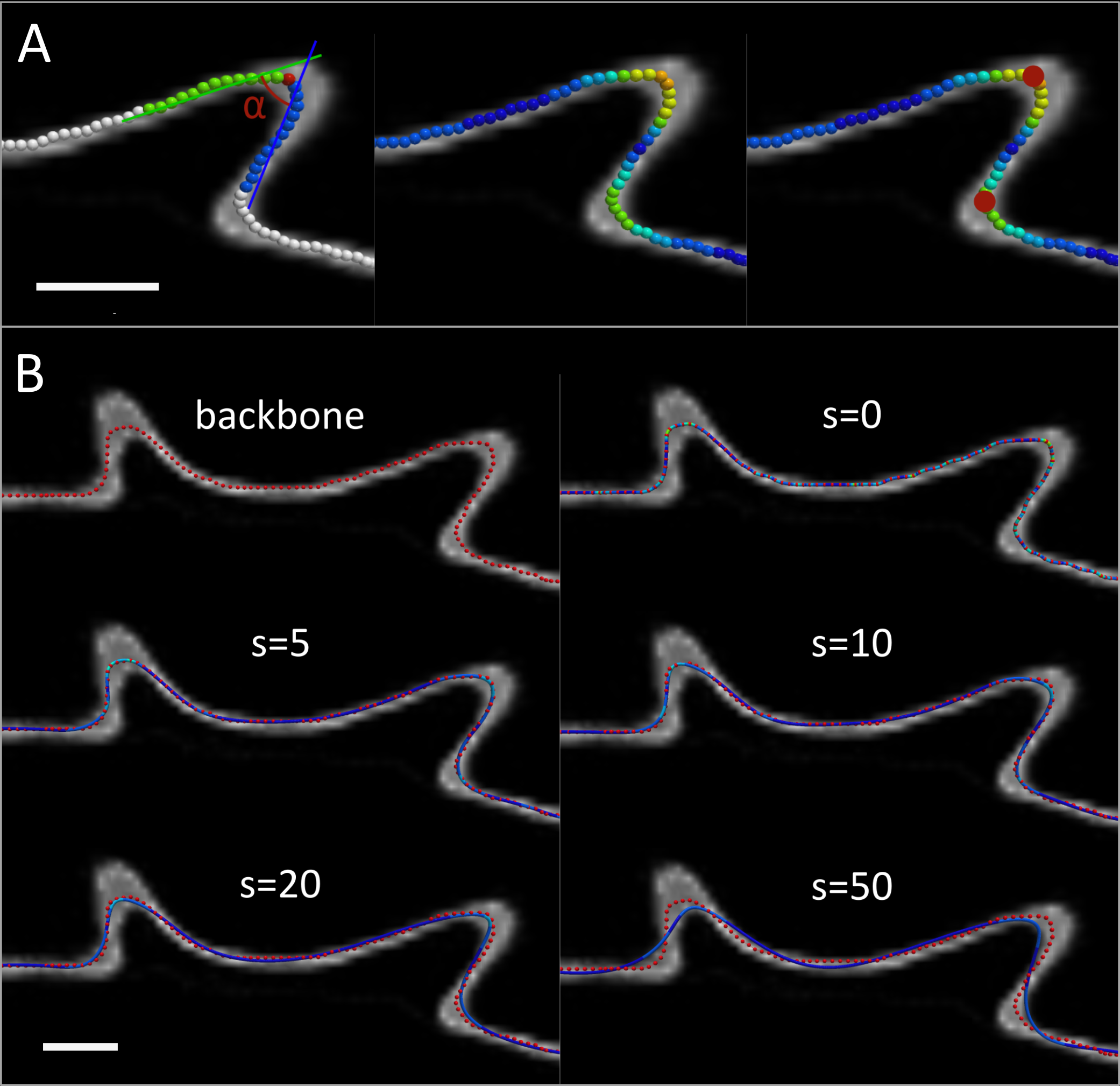

Supplement: Extended Data Figure 1-3 — Two approaches for the quantification of sharp bends. A, left panel, For every node (red) along the main branch, an angle (α) was calculated by linearly approximating upstream and downstream nodes in a window of 2 μm (red and green). Middle panel, Colors represent the angles calculated for every node as specified in the first panel. Blue corresponds to wider angles, orange to narrow angles. Right panel, Nodes with minimum angles were selected sequentially (red dots), double counting was avoided by non-maximum suppression. B, Visualization of the main branch backbone (red) and B-spline approximations of with differing amounts of smoothing (s). Blue colors correspond to parts of low curvature, orange to high-curvature. Scale bar = 2 μm. Download Figure 1-3, TIF file. [file sup_enu-eN-MNT-0014-19-s03.tif]

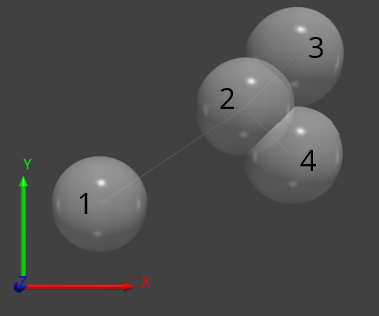

Supplement: Extended Data File 1. — Code and instruction for the neuronal quantification analysis. The zip file includes the python code files for batch processing, beads, classify, clean up, kink positions, soma volume, utility, and waviness. In addition, a swc example image, readme, requirements, and license text files are also included. Figure Contributions: Max Hess acquired confocal images. Max Hess, Alvaro Gomariz, Orcun Goksel, and Collin Ewald devised the image analysis pipeline, which was implemented by Max Hess and Alvaro Gomariz. Max Hess and Collin Ewald wrote the legends in consultation with the other authors. Download Extended Data F, ZIP file. [file sup_enu-eN-MNT-0014-19-s11.zip › NeuronMorphologyQuantification/images/swc_example.png]

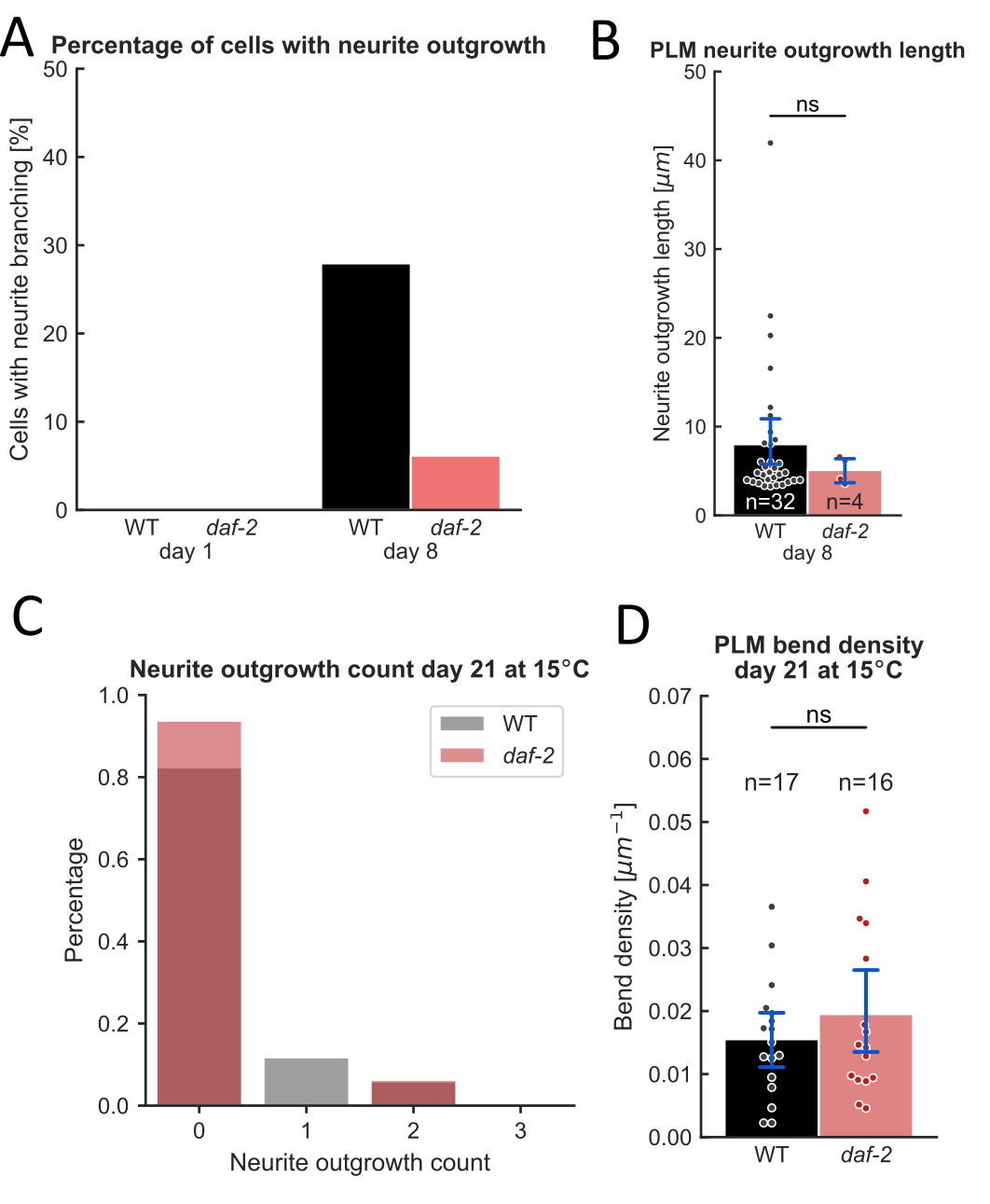

Supplement: Extended Data Figure 3-1 — Comparison of PLM morphological changes of three-week-old C. elegans maintained at lower temperature. A, Percentage of PLM neurons with neurite outgrowths of WT and long-lived daf-2(e1370) at day 1 and day 8 of adulthood at 25°C. Same data as in Figure 3B plotted as percentage to make it comparable to previous studies. B, Measurements of individual neurite outgrowths lengths observed in WT and daf-2 day-8 animals raised at 25°C does not show a significant difference due to the overall low number of observations of neurite branches (especially in daf-2 mutants). C, Neurite outgrowth count of WT and daf-2 animals raised at 25°C for 21 d does not show a significant difference between conditions (p = 0.837; WT n = 17, daf-2 n = 16). D, Bend density of WT and daf-2 animals raised at 25°C for 21 d does not show a significant difference between conditions (p = 0.911). Download Figure 3-1, TIF file. [file sup_enu-eN-MNT-0014-19-s06.tif]

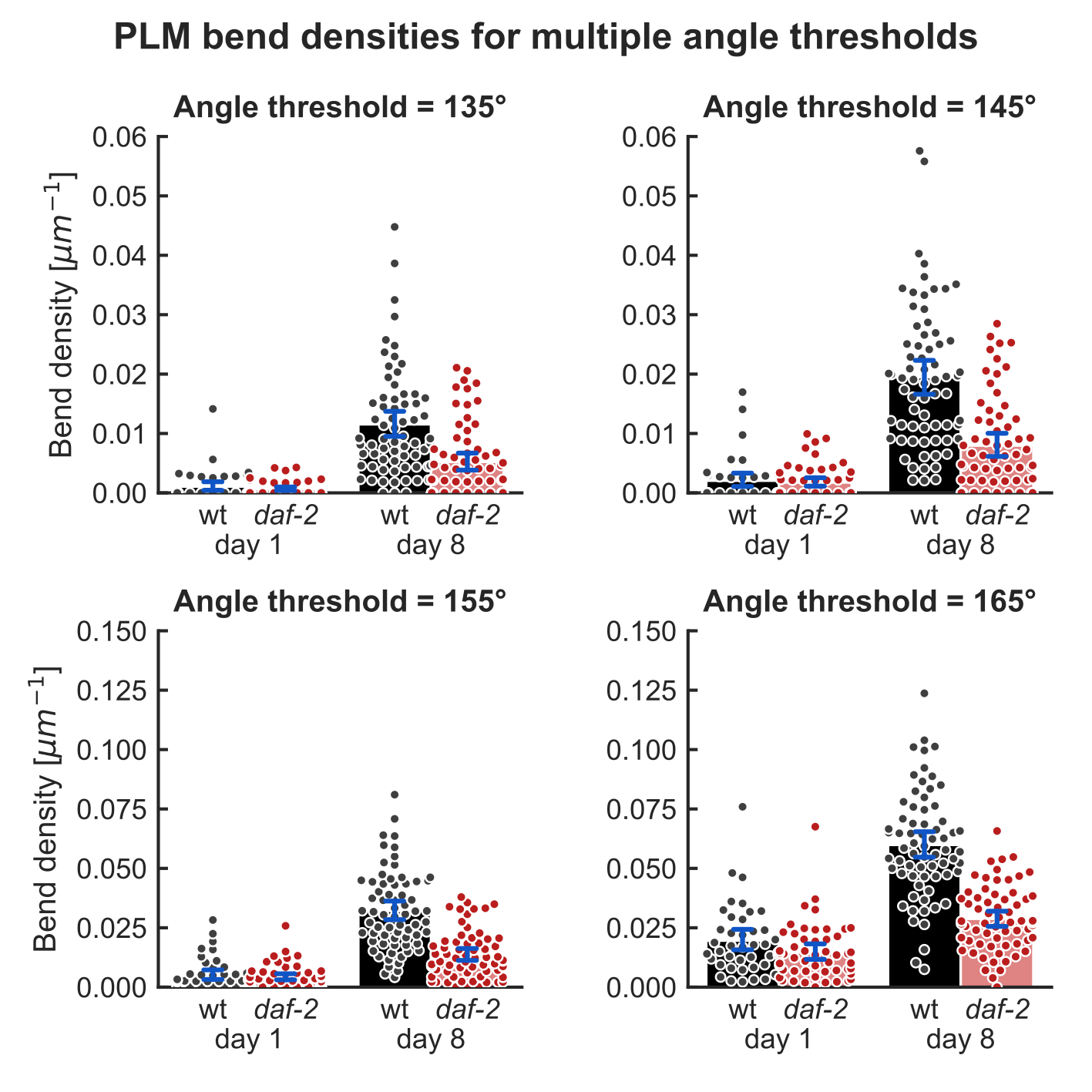

Supplement: Extended Data Figure 3-3 — Robustness of PLM bend densities across different angles threshold settings. Using the data from Figure 3D with different angle thresholds, ranging from 135° to 165°, are quantified and show similar results of bend density across genotype and age. As expected, increasing the threshold leads to higher bend densities, but the differences between conditions are insensitive to changes in threshold angle settings. Dots show individual measurements and blue error bars are bootstrapped 95% confidence interval. Please note, the differently scaled axes between the rows. Download Figure 3-3, TIF file. [file sup_enu-eN-MNT-0014-19-s08.tif]

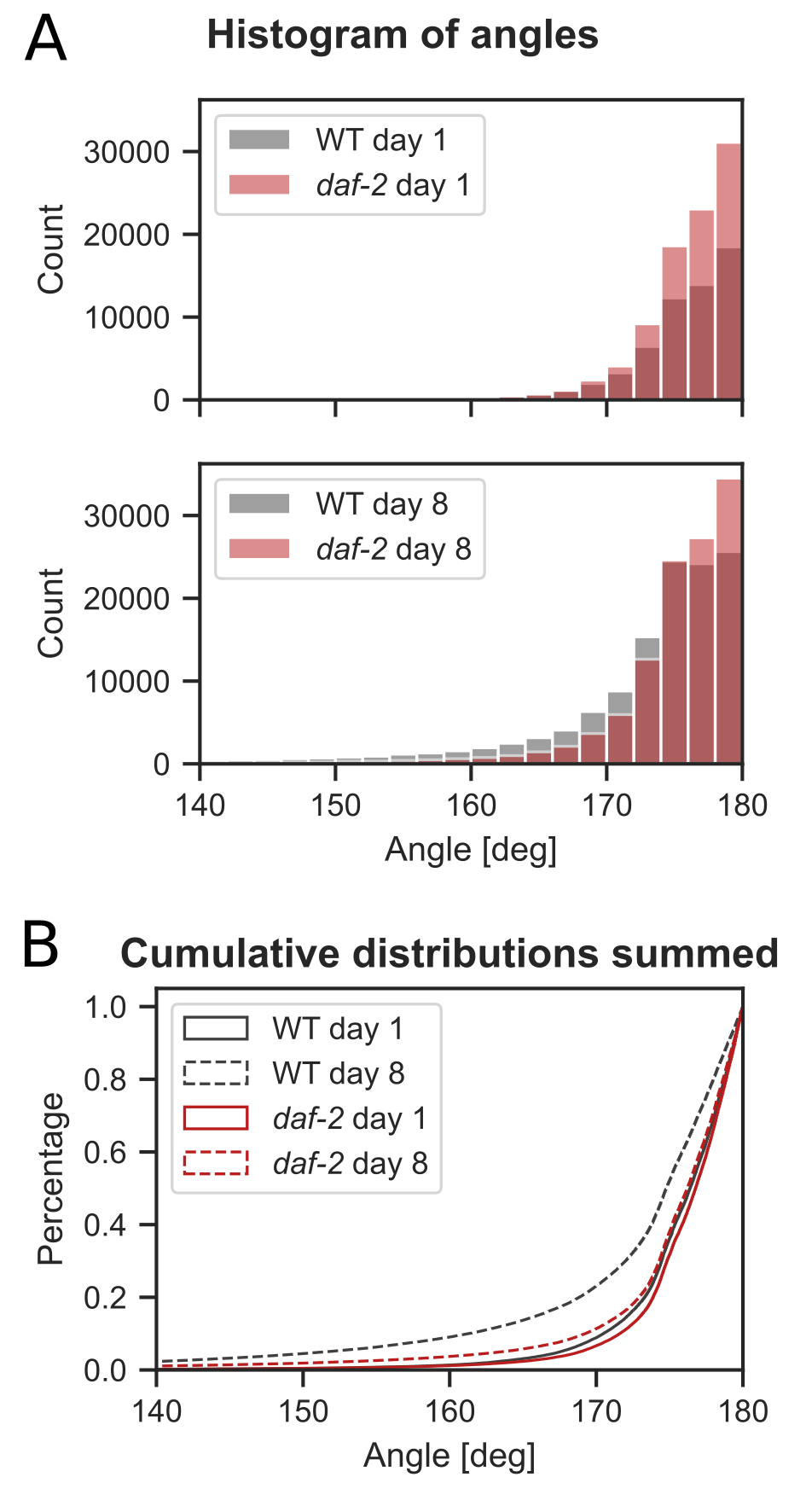

Supplement: Extended Data Figure 3-4 — Histogram and CDFs across angles. Using data from Figure 3D, histogram and cumulative distribution of angles are plotted. A, Histogram showing angle measurements of all PLM main branch nodes for WT and daf-2(e1370) mutants at days 1 and 8 of adulthood. B, Corresponding cumulative distributions of all angles for WT and daf-2(e1370) mutants at days 1 and 8 of adulthood. Download Figure 3-4, TIF file. [file sup_enu-eN-MNT-0014-19-s09.tif]

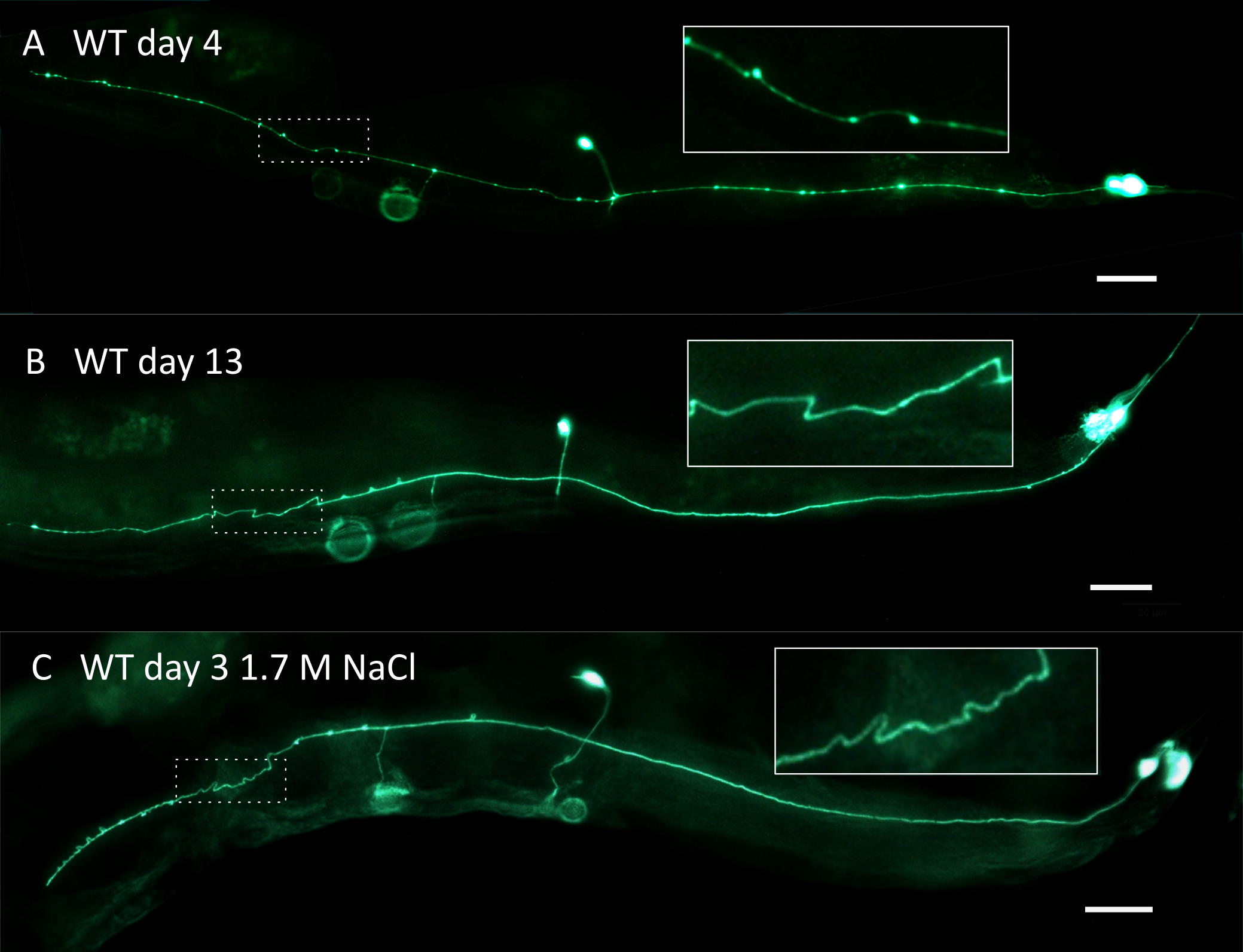

Supplement: Extended Data Figure 3-5 — Osmotic shrinkage of C. elegans results in bending of PLM neurons that look different than the age-related sharp bends. A, Fluorescent image of a typical PLM neuron at day 4 of adulthood shows beads along the process but no sharp bends. B, Fluorescent image of a typical PLM neuron at day 13 of adulthood shows a couple of sharp bends along the process. C, Fluorescent image of a typical PLM neuron at day 3 of adulthood that was imaged upon placing the C. elegans in a drop of 1.7 M NaCl to induce osmotic pressure. The induced wrinkles appear different (rounder curvature as observed when neurons supercoil; Krieg et al., 2017; see Discussion) than the age-related morphology. For A–C, insets are 3× zoom of dashed rectangles. Scale bars = 20 μm. Download Figure 3-5, TIF file. [file sup_enu-eN-MNT-0014-19-s10.tif]
